# Factors affecting the sustainability of community mental health assets: A systematic review

**DOI:** 10.1111/hsc.13929

**Published:** 2022-07-28

**Authors:** Anna Moore, Marcello Bertotti, Ainul Hanafiah, Daniel Hayes

**Affiliations:** ^1^ Evidence Based Practice Unit (EBPU) University College London and Anna Freud National Centre for Children and Families (AFNCCF) London UK; ^2^ Institute for Connected Communities (ICC) University of East London London UK; ^3^ Research Department of Behavioural Science and Health Institute of Epidemiology & Health Care, University College London London UK

**Keywords:** community, health assets, mental health, public health, sustainability

## Abstract

Resources and activities offered by Voluntary, Community and Social Enterprise (VCSE) organisations could play a key role in supporting communities with their mental health. Whilst policy makers have become increasingly interested in using such asset‐based approaches to improve mental health and well‐being, the sustainability of these approaches remains underresearched. In this review, we explored the factors affecting the sustainability of community mental health assets. We conducted a systematic review of the literature using keywords based on three key terms: ‘sustainability’, ‘mental health issues’ and ‘service provision’. Our search strategy was deployed in four electronic databases (MEDLINE, Web of Science, ASSIA and IBSS) and relevant websites were also searched. The literature search was conducted in November and December 2020 and yielded 2486 results. After title and abstract screening, 544 articles were subjected to full‐text review. A total of 16 studies were included in a narrative synthesis. Studies included a broad range of community interventions and 30 factors affecting sustainability were identified across three sustainability levels: micro (individual), meso (organisational) and macro (local/national/global). Factors were discussed as barriers or facilitators to sustainability. A key barrier across all sustainability levels was funding (cost to individual participants, lack of available funding for VCSEs, economic uncertainty) whilst a key facilitator was connectedness (social connections, partnering with other organisations, linking with national public health systems). Nearly all articles included no definition of sustainability and the majority of factors identified here were at the meso/organisational level. As funding was found to be such a prevalent barrier, more research into macro level factors (e.g. government policies) is required.

**What is known about this topic?**

- There is a growing reliance on and need for community assets to support public mental health
- Such community assets may help to address health inequalities and improve mental health outcomes
- Changing social and political influences have resulted in a landscape where the sustainability of community health activities is continuously under threat

**What this paper adds?**

- A synthesis of the available literature and a list of factors affecting sustainability of community mental health assets
- Detailed list of sustainability factors could inform planning for sustainability, both at the organisational and regional/national level
- Recommendations for future research, such as improving definitions and evaluations of sustainability, and exploring cost‐effectiveness of interventions in community settings

## 1 INTRODUCTION

Improving people's mental health and well‐being has been identified as one of the key public health issues of our time, and the global coronavirus pandemic has created even greater need for mental health support (Pieh et al., 2021; Rajkumar, 2020; Vindegaard & Benros, 2020). In recent years, policy makers have become increasingly interested in improving health and well‐being through asset‐based approaches, and multiple studies have shown that participation in community health assets is associated with higher quality of life (Morgan & Ziglio, 2007; Munford et al., 2017; Munford et al., 2020; Van Bortel et al., 2019). However, health assets have been variously defined, ranging from material resources (e.g. land and buildings), individual or collective psychosocial attributes (e.g. skills, capacity, knowledge and passions) and the networking of these to improve the health and well‐being of communities (Foot, 2012; Friedli, 2013; Garven et al., 2016; Munford et al., 2017).

A recent systematic review of the literature on health assets in a global context found that Morgan and Ziglio's (2007) definition of a health asset as ‘as any factor (or resource) which enhances the ability of individuals, groups, communities, populations, social systems and/or institutions to maintain health and well‐being and to help to reduce health inequalities’ was the most frequently cited (Van Bortel et al., 2019). Van Bortel et al. (2019) note the importance of such community assets for health and well‐being. This is pertinent to the UK context, where widening health inequalities are leading to the growth of a range of mental health issues, many of which have been exacerbated by pandemic conditions (Marmot, 2020; Naylor et al., 2012: Suleman et al., 2021). Despite growing investment in mental health (e.g. Improving Access to Psychological Therapies, Big Society), the UK government is still facing an increased demand for mental health support (Cabinet Office, 2015; Suleman et al., 2021). In this context, this research focuses on the resources and activities offered by Voluntary, Community and Social Enterprise (VCSE) sector organisations to support community mental health as a complementary strategy to support good mental health and tackle mental health difficulties (Foot, 2012; Friedli, 2013). Such support extends to a variety of non‐clinical support for mental health and well‐being such as leisure, social connection, education and the arts (Munford et al., 2017).

Despite providing key support to local populations in the UK, the financial sustainability of community health interventions is continuously under threat as a result of a dependence on government grants. Between 2009 and 2019, local authorities experienced 38% cuts in central government grants which has led to the significant decline of community health interventions (Institute for Government, 2021). The sustainability of these types of health asset is very important for the delivery of mental health interventions at the community level; the more community‐based approaches can be sustained, the better the health and well‐being of individuals and communities is likely to be. Thus, the investigation of factors affecting sustainability is important in maximising the potential impact of community health interventions.

Whilst there is a broad literature on sustainability of public health programmes, it remains fragmented and underdeveloped (Schell et al., 2013; Wiltsey Stirman et al., 2012). Many studies exploring long‐term delivery of interventions and activities provide no definition of sustainability, and there is also a lack of consensus around core constructs (Schell et al., 2013; Wiltsey Stirman et al., 2012). In their review, Wiltsey Stirman et al. (2012) found four overarching factors influencing sustainability across literature on healthcare, mental health studies and public health/health promotion. These were innovation characteristics (e.g. fit, effectiveness, ability to be modified), context (e.g. climate, leadership, system/policy change), processes and interactions (e.g. shared decision‐making amongst stakeholders, evaluation and feedback, planning, collaboration) and capacity (e.g. champions, funding, resources). In the field of public health, Schell et al. (2013) aimed to identify the core domains that affect a programme's capacity for sustainability and drew them together in a conceptual framework. The nine domains identified are similar to those found in Wiltsey Stirman et al.'s (2012) review: Political Support, Funding Stability, Partnerships, Organizational Capacity, Program Evaluation, Program Adaptation, Communications, Public Health Impacts and Strategic Planning (Schell et al., 2013). Whilst these factors may also apply to community mental health assets, it is important to understand sustainability in the specific context of resources and activities offered by VCSE organisations.

The growing importance of health assets for policy makers outlined above, along with the limited research on community assets for mental health and the importance of sustainability for such assets, reveals an important gap in the literature. In order to address this, we conducted a systematic review to answer the following question: what factors affect the sustainability of community mental health assets? Through this review, we aimed to identify policy priority areas that may support sustainability of these assets and to highlight gaps in current research.

## 2 METHODOLOGY

Amongst the types of systematic reviews that can be undertaken the ‘systematic map’ is useful to describe the existing research literature on a broad topic area (i.e. sustainability and community mental health assets; Gough et al., 2017). In undertaking this, we followed the guidance from SCIE (Clapton et al., 2009) and registered this review with PROSPERO (CRD42021233171).

### 2.1 Inclusion and exclusion criteria

In order to explore this research question, we developed a range of inclusion and exclusion criteria (see Table 1 for details) around the key constructs of this research including ‘mental health or mental well‐being’, ‘community assets’ (Garven et al., 2016; Munford et al., 2020) and factors affecting ‘sustainability’. Of the numerous definitions and frameworks used to conceptualise the term ‘sustainability’, Wiltsey Stirman et al. (2012) found that the most commonly cited definition in the literature was proposed by Scheirer (2005). This defines sustainability on three different levels: (i) Individual Level: continuing to deliver the desired outcomes or benefits for individual community members; (ii) Organisational Level: an organisation maintaining the programme or intervention in an identifiable form, even if modified; (iii) Community Level: maintaining the capacity of a community/region/nation to deliver programme activities after an initial implementation period is over (Scheirer, 2005). In this paper, we drew on this definition and searched for papers discussing sustainability at any of these levels.

**TABLE 1:** Inclusion and exclusion criteria

Key concept	Inclusion criteria	Exclusion criteria
Mental health or mental well‐being	Mental health/well‐being is one of the outcomes being evaluated *OR* An intervention is targeted at a specific population of people with mental health difficulties (e.g. increasing exercise frequency in people with severe mental health problem)	Outcomes relate to physical health only Intervention is targeted at neurodivergent populations (e.g. autism, dyslexia) with no mention of mental health
Community assets	Interventions delivered in community settings (e.g. youth groups, volunteer projects, libraries, peer support groups)	Interventions taking place in non‐voluntary public services (e.g. prison, hospitals, schools, inpatient mental health services) Interventions taking place in a private workplace Digital interventions completed by individuals at home on their own
Sustainability	Discussion of factors affecting sustainability at any level (individual/organisational/community)	Long‐term or sustainability follow‐up relates only to individual outcomes (with no information about factors affecting sustainability)

**FIGURE 1:**
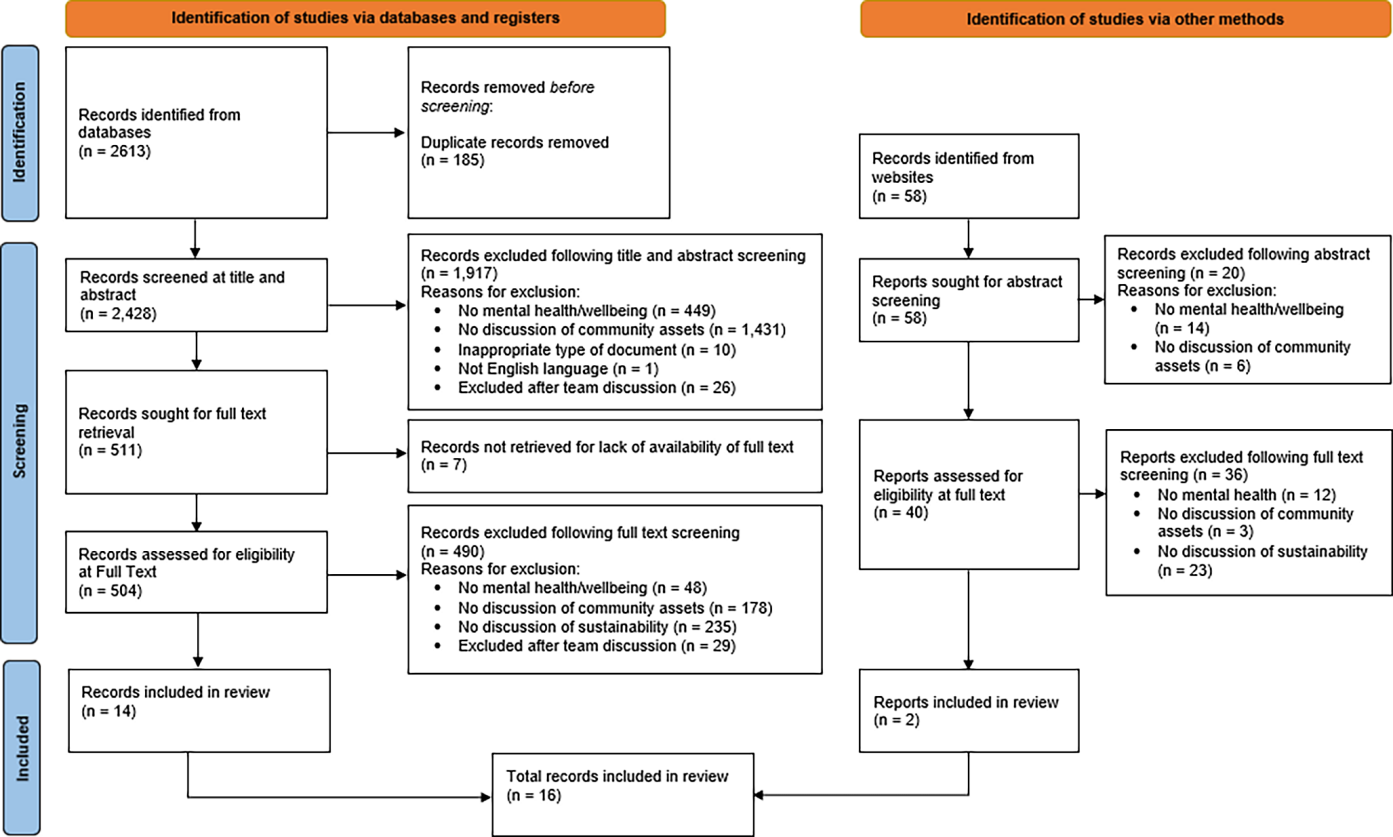
PRISMA flow diagram of included studies (Page et al., 2021).

We used the ‘mental health or mental well‐being’ and ‘community assets’ inclusion and exclusion criteria at the Title and Abstracts screening stage. We then introduced the ‘sustainability’ criteria at the Full‐Text screening, as discussions concerning this theme were less likely to be mentioned in the title or abstract of each article and were instead expected to require reading the full document (see Figure 1 flow diagram for details).

### 2.2 Search strategy

With the support of an information specialist, we identified a wide range of keywords based on the key terms: ‘sustainability’ (e.g. maintenance, durability), ‘mental health issues’ (e.g. anxiety, depression) and ‘service provision’ (e.g. interventions, programmes, therapies). A full list of these terms can be found in Appendix S1. These keywords and associated combinations were searched in the following databases for the period 2010–2020: MEDLINE (Ovid), Web of Science, ASSIA Social Care Online (mental health and community) and IBSS. We also searched a number of relevant websites for grey literature (e.g. King's Fund, Mind, Wellcome Collection) and a full list of websites can also be found in Appendix S1. All searches were carried out between November 1 and December 20, 2020.

### 2.3 Screening

All identified studies were imported into the data management software EPPI‐Reviewer Web (Thomas et al., 2020). A two‐stage process was undertaken for screening. The first stage involved screening article titles and abstracts, during which all reviewers (AM, DH, MB and CF) independently screened the same 5% of records. The remaining records were then split between the reviewers and screened on title and abstract. Full‐text copies of the remaining articles were retrieved, and all four reviewers again screened an initial 5% before meeting to finalise inclusion and exclusion criteria. Each reviewer then independently screened a portion of the full texts. The second stage consisted of full‐text screening in which the same process was repeated. Studies where there was uncertainty were discussed in the research team until a consensus was reached.

### 2.4 Quality assessment

Two authors (AM and AH) conducted quality assessment of the included articles, using the Mixed Methods Appraisal Tool (MMAT; Hong et al., 2018). This tool, designed to help appraise the methodological quality of research studies, allows for simultaneous evaluation of all empirical literature (i.e. qualitative, quantitative and mixed methods studies), which was deemed appropriate for this review. The MMAT has high intraclass correlation and has been shown to be efficient and user‐friendly (Pace et al., 2012). Both AM and AH independently scored all of the included articles and then met to finalise the scores. Quality scores for each article ranged from ‘low’ meeting none of five criteria (zero) to ‘high’ meeting all five criteria (five).

### 2.5 Data extraction

A data extraction table was designed by the study team specifically for this review, drawing on best practice guidance (Centre for Reviews and Dissemination, 2008). Extracted variables included: geographical location; study aim and design; study population; description of community intervention; data collection methods; and sustainability definition and factors. Extracted sustainability factors included barriers and facilitators at any level included in Scheirer's (2005) definition. We decided, however, to use the terms micro, meso and macro here (instead of Scheirer's (2005) individual, organisational and community levels) as we wanted to convey the breadth of the highest level (macro) in this work, which covers regional and national influences rather than only the immediate community.

### 2.6 Data synthesis

A narrative synthesis (Popay et al., 2006) was used to provide a critical evaluation of evidence on factors affecting sustainability of community assets for mental health, examining factors at the micro, meso and macro levels. AM led on the data synthesis, coding line‐by‐line data from the included studies that discussed factors affecting sustainability. Preliminary factors were created and shared with the study team. These were refined through discussion and then split into the three sustainability levels (micro, meso and macro). The narrative synthesis examined similarities and differences across the levels and explored overarching themes across the studies.

## 3 RESULTS

### 3.1 Search results

As depicted in Figure 1, database and wider website searches returned 2486 records and the first stage of screening (titles and abstracts) resulted in the exclusion of 1899 records (see Table 1 and Section 2.1 for exclusion criteria). The second stage of screening (full text) resulted in the exclusion of 528 records. In total, 16 articles were identified that met inclusion criteria and provided extractable information on factors affecting sustainability.

### 3.2 Study characteristics

The countries with the most included articles, both with five studies, were the United States (Donnelly et al., 2020; Ferré et al., 2010; Fleisher et al., 2020; Gorman et al., 2018; Palinkas et al., 2019) and the United Kingdom (Foster et al., 2020; Grant et al., 2017; Kelly et al., 2019; MHF, 2017, 2018). The remaining studies were based in China (Fan et al., 2018), Denmark (Martin et al., 2012), India (Shields‐Zeeman et al., 2017), Mongolia (Witte et al., 2019), Pakistan (Atif et al., 2019) and Spain (Coll‐Planas et al., 2017).

Studies presented findings from a range of different community‐based interventions, including: those focused on physical activities such as yoga, karate and walking (Donnelly et al., 2020; Fleisher et al., 2020; Grant et al., 2017); peer support and community volunteer interventions (Atif et al., 2019; Fan et al., 2018; MHF, 2017, 2018; Shields‐Zeeman et al., 2017); those targeting social isolation (Coll‐Planas et al., 2017; Foster et al., 2020; Kelly et al., 2019); and wider interventions for specific community groups (Ferré et al., 2010; Gorman et al., 2018; Martin et al., 2012; Palinkas et al., 2019; Witte et al., 2019).

### 3.3 Defining sustainability

Only one article discussed different conceptualisations of sustainability, highlighting current uncertainty in the literature regarding definitions and exploring the idea of sustainability as a process, an outcome or both (Palinkas et al., 2019). Four studies referred to sustainability as an activity being maintained (Gorman et al., 2018; MHF, 2018; Shields‐Zeeman et al., 2017; Witte et al., 2019) and one study referred to the scaling‐up of the intervention (Atif et al., 2019). The remaining 10 studies provided no definition of sustainability.

### 3.4 Quality assessment

Quality of the studies was generally good (scores on the MMAT of 4 to 5), although some of the mixed methods studies did not fully integrate qualitative and quantitative components or address any divergences and inconsistencies between the two (Fan et al., 2018). Two of the included studies could not be assessed using the MMAT as they were ‘lessons learned’ pieces and not empirical studies (Ferré et al., 2010; Witte et al., 2019). A summary of study design, data collection methods, intervention characteristics and quality appraisal is provided in Table 2.

**TABLE 2:** Description of articles in the review

Article number	Author, year of publication, country	Study design	Data collection methods	Targeted population of intervention	Intervention aim	Intervention description/components	Intervention deliverer(s)	Quality assessment score
1	Atif et al., 2019, Pakistan	Convergent mixed methods	Focus groups, questionnaires	Women with perinatal depression	To reduce severity of depressive symptoms and prevalence of remission	Individual and group sessions, based on principles of cognitive behavioural therapy	Peer volunteers (local women), alongside community health workers	4
2	Coll‐Planas et al., 2017, Spain	Quantitative – cohort study	Telephone questionnaires, interviews	Older adults, self‐referred or professionally referred for loneliness	To alleviate loneliness and improve health (including quality of life and depressive symptoms)	Group‐based programme and coordinated action (building and strengthening network between healthcare centres, senior centres and other community assets)	Group facilitator (e.g. nurse or social worker) and volunteers	4
3	Donnelly et al., 2020, USA	Qualitative – phenomenology	Interviews	People with traumatic brain injury and their carers	To promote community integration	Yoga, meditation techniques and psychoeducation	Specifically trained yoga teachers	5
4	Fan et al., 2018, China	Convergent mixed methods	Interviews, questionnaires	Adults with severe mental health problem	Peer support model to assist with providing rehabilitation services	Peer‐led activities focused on topics such as daily life skills, knowledge of mental disorders and emotional support	Peer service providers (with a previous diagnosis of schizophrenia or bipolar disorder)	2
5	Ferré et al., 2010, USA	Lessons learned	Reflections from research team	African‐American families	To improve the health outcomes in African‐American communities	Community assets model incl. Research and evaluation, networking, community education, needs assessments, training	Organisation led by Executive Director and a governing body	n/a – not empirical study
6	Fleisher et al., 2020, USA	Quantitative – non‐randomised control trial	Questionnaires	Adults with mild to moderate stage Parkinson's Disease	To offer positive effects on mobility, balance and quality of life	Community‐based, structured, non‐contact karate classes	Karate instructors	4
7	Foster et al., 2020, UK	Convergent mixed methods	Interviews	Adults referred to a social prescribing service to address loneliness	To reduce loneliness	Social prescribing services incl. Craft groups, adult learning and leisure facilities	Social prescribing link workers	4
8	Gorman et al., 2018, USA	Convergent mixed methods	Interviews, questionnaires	Adults and young adults with serious mental health problem	To support people back into the community (help people meet their needs for health, education, social connections and housing)	Clubhouses – voluntary psychosocial rehabilitation	Clubhouse staff and members	4
9	Grant et al., 2017, UK	Qualitative – ethnography	Interviews, ethnography	Walking group for adults with long‐term health conditions	To improve health and well‐being	Two walking groups (one for shorter walks and the other for longer distances)	Volunteer walk leaders	5
10	Kelly et al., 2019, UK	Qualitative – description	Interviews	People experiencing social isolation and loneliness in rural communities	To reduce social isolation and loneliness	Variety of social enterprises incl. Community hub, education centre, community café	Social enterprise staff and volunteers	5
11	Martin et al., 2012, Denmark	Convergent mixed methods	Focus groups, questionnaires	Adults on long‐term sickness absence from work due to common mental health problems	To facilitate an early return to work and reduce sickness absence and symptoms of mental health problems	Return to work plans incl. sessions with clinicians, planning of daily activities and individual‐ and group‐based courses (e.g. stress management, conflict resolution, relaxation training)	Private company specialising in a coordinated and tailored return to work approach	5
12	Mental Health Foundation (MHF), 2017, UK	Convergent mixed methods	Interviews, focus groups, questionnaires	Single parents	To help develop skills and build support networks that enable single parents to maintain their health and well‐being	Group sessions incl. mental health awareness, relaxation and mindfulness techniques, goal‐setting, peer support	Charity workers and volunteers	3
13	Mental Health Foundation (MHF), 2018, UK	Convergent mixed methods	Interviews, questionnaires	Young mothers	To enhance well‐being of young mums and their children	Drop‐in sessions incl. creative activities, opportunities for play, interactive discussions and guest speakers	Peer support volunteers	4
14	Palinkas et al., 2019, USA	Qualitative – description	Interviews	STOP Act – youth and young adults at risk of alcohol abuse; Garrett Lee Smith Suicide Prevention – youth and adults	To minimise alcohol abuse; to address substance abuse and other health problems (e.g. depression) with risks directly linked to suicide	Grant funding provided to 53 states, tribes and territories, required funds be used by grantees for programme development	*Not reported*	5
15	Shields‐Zeeman et al., 2017, India	Qualitative – case study	Focus groups, interviews	People with mental health problems in a community in rural India	To provide support and basic counselling to community members, facilitate access to care, improve community awareness of mental health issues, promote well‐being	Promoting healthy lifestyle behaviours, counselling sessions	Community volunteers	4
16	Witte et al., 2019, Mongolia	Lessons learned	Reflections from research team	Women engaged in sex work	To reduce risk for transmission of STIs and improve mental health	Group sessions based on social cognitive theory, skills targeted to increasing self‐efficacy and risk reduction, also microfinance and financial literacy sessions	*Not reported*	n/a – not empirical study

### 3.5 Factors affecting sustainability

For a list of the factors affecting sustainability that were discussed in each article see Table 3. Results are organised here according to the three sustainability levels outlined the methods (Scheirer, 2005): (i) Micro level: factors related to individual participants continuing to receive the desired outcomes or benefits of the intervention; (ii) Meso level: factors related to an organisation's ability to maintain the programme or intervention in an identifiable form; (iii) Macro level: factors related to the capacity of a community/region/nation to continue delivering programme activities after the end of an initial implementation period.

**TABLE 3:** Factors affecting sustainability

Sustainability level^a^	Factors – Key themes	Factors ‐ subthemes	Atif et al. (2019)	Coll‐Planas et al. (2017)	Donnelly et al. (2020)	Fan et al. (2018)	Ferré et al. (2010)	Fleisher et al. (2020)	Foster et al. (2020)	Gorman et al. (2018)	Grant et al. (2017)	Kelly et al. (2019)	Martin et al. (2012)	Mental Health Foundation (2017)	Mental Health Foundation (2018)	Palinkas et al. (2019)	Shields‐Zeeman et al. (2017)	Witte et al. (2019)
Micro (individual)	Funding	Cost to participants						−	−		+/−							
	Intervention	Logistics	−						−					−	−			
		Perceived benefit	+	+	+			−		+	+					+		
		Social connections	+	+	+						+			+				
Meso (organisational)	Funding	Availability of funding	−				−					−			−	+/−		+/−
		Diversity of funding								+/−								
		Skills/knowledge/capacity to apply for funding								+/−		−						+
		Financial support for volunteers	+/−												−			
	Strategy	Sustainability planning												+		+		+
		Progress monitoring processes								+				+				
		Community partnered participatory research (CPPR)					+											
	Intervention	Fit with organisational culture and values														+		
		Flexibility and adaptability of intervention		+							+		+	+				
		Access to resources												+/−			+	
		Evidence of positive outcomes/meeting needs											+					
		Engagement from community participants	+/−				+/−							+/−	+/−			
	Staffing/personnel	Clear roles and responsibilities				+											+	
		Workforce morale and burnout	−									−						
		Staff/volunteer turnover												−	−			
		Capacity to pursue sustainability														+/−		−
		Leadership involvement and support				+				+/−				+				
		Programme champions					+									+		
		Resilience, commitment and resolve of staff	+				+			+							+	
		Opportunity for progression	+											+				
		Supervision and training	+			+												
Macro (local, regional, national, global)	Funding	Funding priorities									−			−				−
		Economic uncertainty									−			−				
	Strategy	Partnering with external organisations											−		+			
		Involvement in regional/national initiatives																+
	Intervention	Linking to wider public health systems														+	+	

*Note*: ‘+’ = facilitator, ‘−’ = barrier, ‘+/−’ = discussed as both a barrier and a facilitator.

^a^ Based on Scheirer's (2005) sustainability levels: micro (beneficiary), meso (VSCE organisations), macro (local/regional/national/international).

#### 3.5.1 Micro level factors (individual)

Some of the most prominent factors at the individual level were perceived benefit of the intervention and the opportunity to form social connections (Atif et al., 2019; Coll‐Planas et al., 2017; Donnelly et al., 2020; Gorman et al., 2018; Grant et al., 2017; MHF, 2017; Palinkas et al., 2019). Participants experiencing increased satisfaction and well‐being, learning new techniques or skills, and establishing and maintaining social contacts were all described as facilitators to the sustainability of community interventions. Only one study referenced a perceived lack of benefit as a barrier at the individual level (Fleisher et al., 2020).

Logistical challenges were described as key barriers to sustainability, with participants in some cases struggling to fit the intervention into their week or unable to attend sessions at specific times (MHF, 2017). Transport to where the intervention takes place (and sometimes the associated financial burden placed on participants) was also a barrier, along with the difficulty of using and managing local venues (Atif et al., 2019; Fleisher et al., 2020; Foster et al., 2020; MHF, 2017, 2018). In contrast, one study highlighted the benefit of a local walking group as an intervention with minimal barriers to participation (Grant et al., 2017).

#### 3.5.2 Meso level factors (Organisational)

Availability of funding and staff skills and capacity to make grant applications were key factors for community organisations. Several articles cited limited funding as a strong barrier to sustaining activities (Atif et al., 2019; Ferré et al., 2010; Kelly et al., 2019; Witte et al., 2019), whilst one study also highlighted the danger of relying too heavily on one funding source (Gorman et al., 2018). Kelly et al.'s (2019) study on social enterprises found that staff and volunteers lacked the confidence and skills to write successful grant applications, whereas others discussed success in securing ongoing funding when teams had capacity and the right support (Gorman et al., 2018; Witte et al., 2019).

High staff and volunteer turnover, combined with low morale and burnout, also posed considerable issues for sustainability at the organisational level. These barriers were heightened in certain contexts, for example, in remote or rural populations (Kelly et al., 2019) and where there was uncertainty about the programme's future (Atif et al., 2019; MHF, 2018). Two studies described the benefits of planning for financial support for peer volunteers, stating that small amounts to help with the running of group sessions (e.g. food, craft materials) can serve as an incentive or may be a barrier if groups are not able to offer a consistent level of activities and resources (Atif et al., 2019; MHF, 2018).

Engagement from the local community or target population was both a facilitator in some instances and a barrier in others (Atif et al., 2019; Ferré et al., 2010; MHF, 2017, 2018). An example of engagement as a barrier is described in Atif et al.'s (2019) study, when low attendance led to volunteers feeling helpless and demotivated. In contrast, the positivity, commitment, resilience and resolve of staff were all cited as facilitators to sustaining activities, along with the opportunity for volunteers to progress and develop new skills (Atif et al., 2019; Fan et al., 2018; Ferré et al., 2010; Gorman et al., 2018; MHF, 2017; Palinkas et al., 2019; Shields‐Zeeman et al., 2017). Staff and volunteers were described as working best when there were clear responsibilities laid out from the start (Fan et al., 2018; Shields‐Zeeman et al., 2017).

Another facilitator was the flexibility and adaptability of the intervention at varying levels, from small‐scale walking groups that can be adapted for participants' fitness levels to wider interventions making use of Community Partnered Participatory Research (CPPR) to build a programme of activities (Ferré et al., 2010; Grant et al., 2017). A number of studies also highlighted the importance of planning for sustainability at the early stages of a project or intervention, along with allocated staff time for sustainability work, regular progress monitoring and the involvement of VSCE organisation leadership (Fan et al., 2018; Gorman et al., 2018; MHF, 2017; Palinkas et al., 2019; Witte et al., 2019).

#### 3.5.3 Macro level factors (local/regional/national/global)

Funding and economic uncertainty were the most cited factors affecting sustainability at the higher level, including shifting research priorities, austerity and an over‐reliance on voluntarism in public health systems (Grant et al., 2017; MHF, 2017; Witte et al., 2019). Facilitators to sustainability include working closely with regional or national public health systems and partnering with external organisations, although successful cooperation requires good communication and can be detrimental if not done well (Martin et al., 2012; MHF, 2018; Palinkas et al., 2019; Shields‐Zeeman et al., 2017; Witte et al., 2019).

## 4 DISCUSSION

In the current climate, with the persistence of health inequalities, and the importance of community assets for improving quality of life, the aim of this research was to review systematically the factors affecting sustainability of community mental health assets. We aimed to examine how community assets can provide further support to tackling mental health issues, support that could complement the current delivery of statutory mental health services. The search retrieved articles on a broad range of international community interventions, which in turn include a range of barriers and facilitators to sustaining community activities or services. Whilst we found a number of factors at the micro and macro levels, the majority of sustainability factors discussed in the included articles were found at the meso level referring to the sustainability factors of organisations.

Some of the themes identified here recurred across levels. The idea of connectedness occurred in each of the three sustainability levels, from individual participants emphasising the importance of social connections, to the use of methods such as Community Partnered Participatory Research (CPPR) and, at the macro level, the importance of partnering with external organisations (Atif et al., 2019; Coll‐Planas et al., 2017; Ferré et al., 2010; MHF, 2018). Authors emphasised the benefits of linking with local and national public health systems and establishing common ways of working with the staff of state mental health services (MHF, 2018; Shields‐Zeeman et al., 2017; Witte et al., 2019). These findings are in line with previous reviews by Wiltsey Stirman et al. (2012) and Schell et al. (2013), which suggest that core enablers in the sustainability of public mental health programmes are collaboration, partnerships and linking into wider systems. However, in the slightly different context of school mental health programmes, a recent review on sustainability did not find partnerships and wider systems to be such a prevalent factor (Moore et al., 2022). Reasons for this difference may lie in funding structures for core versus additional staff; schools have greater ‘core’ staff resources to facilitate delivery that are not as affected by hostile funding climates where limited, competitive funding is available. In the case of community assets, policy makers and those evaluating interventions should take note of this and plan for ways to link into wider systems prior to starting the intervention.

As a review of asset‐based approaches noted, the term connectedness at the micro individual level also refers to the involvement of service users in the design, implementation and evaluation of community interventions as a way to encourage greater sustainability at the micro/individual level (Hopkins & Rippon, 2015). The World Health Organization and a number of countries, including the UK government, have promoted the involvement of service users in the design of mental health services. However, more progress has been made in terms of involving individuals in decisions around their personal health rather than at the wider organisational level (Cheng et al., 2017; Storm et al., 2011). It is also important to note that there are several levels of involvement of service users ranging from manipulation to citizen control (Arnstein, 1969). Increasing sustainability for community assets therefore requires greater attention to ensure that service users’ views and opinions are valued. Importantly, this should be meaningful, extending beyond ‘tokenism’ in the involvement of service users to genuine participation (Ocloo & Maathews, 2016; Rutter et al., 2004).

Funding was found also to be a factor affecting sustainability at all levels, highlighting the importance of financial security and continued support for these types of community health assets. At the micro level, individual participants struggled with the cost of travelling to attend activities, whilst at the meso level, limited funding was described as a key barrier to sustaining VSCE programmes (Atif et al., 2019; Ferré et al., 2010; Fleisher et al., 2020; Kelly et al., 2019; Witte et al., 2019). The lack of available funding was found to limit organisational activities and also had an impact on staff capacity, as already‐stretched staff were required to redirect energy to applying for bids and grants (MHF, 2017; Witte et al., 2019). In some instances, the uncertainty around future funding was also found to have a negative effect on workforce morale (Atif et al., 2019). At the highest level, global funding priorities were cited as a factor affecting sustainability, with Witte et al. (2019) referencing severe downsizing of certain activities due to shifting global research priorities. Also at the macro level, economic uncertainty and the impact of austerity policies in certain countries emerged as key factors affecting sustainability (Grant et al., 2017; MHF, 2017).

The role of funding in the sustainability of interventions is consistent with previous reviews (Schell et al., 2013; Wiltsey Stirman et al., 2012). Many community assets depend on their financial sustainability from local authorities, and in the UK, local authorities have seen their funding decline by 38% in the last 10 years (IfG, 2021). However, current government plans (Department for Levelling Up, Housing and Communities, 2021) to develop a ‘Levelling up’ agenda which includes greater devolution of powers to local authorities may lead to positive change. ‘Levelling up’ may provide the right environment for local authorities to employ joined‐up strategies across parts of the councils (e.g. housing, employment, crime) which are crucial to tackle the social determinants of health and mental health. It remains to be seen whether greater autonomy will be accompanied by greater funding transfer to local areas.

In their framework for the sustainability of public health programmes, Schell et al. (2013) synthesised a number of factors that may be related to a programme's ability to sustain its activities and benefits over time. Many of the factors in their framework align with the findings of this review; funding stability, partnerships, programme adaptation and evaluations, organisational capacity, impact and strategic planning all map onto the factors identified in this research. The only factors from the framework not amongst our findings are political support and communications (strategic dissemination of programme outcomes and activities). With the vast majority of articles in this review focusing on organisational factors such as high staff turnover (Atif et al., 2019; Kelly et al., 2019; MHF, 2018) and staff confidence and skills (Gorman et al., 2018; Witte et al., 2019) rather than local authority or national factors, it is perhaps not surprising that these were not identified. However, with the rollout of Integrated Care Systems in the UK and Europe resulting in a sharpened focus between community assets and public healthcare systems (Baxter et al., 2018), it will be interesting to see if these factors become more prominent as a result of the shift in landscape.

## 5 STRENGTHS AND LIMITATIONS

### 5.1 Strengths

This review used a broad definition of sustainability (Scheirer, 2005) and therefore enabled an exploration of factors influencing sustainability at all levels in society, from individual participants (micro) to VSCE organisations (meso) and regional, national and international policy (macro). Through its search strategy, this review captured a wide range of different types of community mental health asset, from small‐scale interventions such as exercise groups, to far broader community interventions with multiple activities. This allowed for an exploration of sustainability for a range of different types of intervention.

This research also focussed on mental health outcomes and interventions, an area of public health that is currently under significant pressure. Consequently, identifying factors that affect sustainability of the work of VSCE organisations could be an important contribution to help alleviate pressure on the system.

### 5.2 Limitations

Whilst an information specialist was utilised and a broad and inclusive approach was taken to the search terms for the construct of sustainability, it is possible that some records were not picked up in the search strategy and thus not included in this review. Additionally, not all records were double screened and we limited the articles in this review to those published in English, excluding potentially relevant studies that may have been published in other languages.

This research was conducted during the first year of the COVID‐19 pandemic, one of the most disruptive global events in recent years (Di Gessa et al., 2021). However, none of the included studies were published after the start of the pandemic and consequently, its effect on community assets is not discussed. This was likely due to the scope of this research and the time taken for academic literature to be produced. Future research should further investigate the role the pandemic has played on the sustainability of community assets.

This research also focussed on a specific type of ‘health asset’ by examining interventions delivered by VCSE sector organisations. However, as highlighted in the introduction, ‘health assets’ are not only confined to VCSE sector organisations but can include physical infrastructure (e.g. libraries), social networks and psychosocial attributes (e.g. individual skills). We could not find a way to include all of these potential definitions of ‘health assets,’ and thus, this is a limitation of our research. However, it is important to note that in the literature, others do refer to health assets as interventions delivered by voluntary sector organisations (e.g. Munford et al., 2020).

## 6 RECOMMENDATIONS

In line with Wiltsey Stirman et al.'s (2012) review on the sustainability of interventions, we found only one article that addressed theories or different conceptualisations of sustainability. We recommend that future studies define sustainability and draw on implementation and sustainability frameworks to shape their research. The list of sustainability factors provided by this research could form the starting point for the development of a framework specific to community mental health assets (see also Palinkas et al.,  2019). It is also recommended that any researchers looking to evaluate sustainability plan their evaluation from the start of the project and think carefully about measures and data collection methods. In many cases, commissioners require intervention providers to evaluate the impact of their community mental health interventions on health and well‐being. Such evaluations could be strengthened by including evaluations of the sustainability factors identified in this study.

Whilst Scheirer's (2005) work explores sustainability as an outcome, where benefits, activities or workforce capacity are maintained, others have suggested that this linear perspective on sustainability ‘does not take account of the recursive or reflexive character of sustainability and learning or of the continuous adjustments that shape the sustainability process’ (Pluye et al., 2004, p. 124). Similarly, Lennox et al. (2018) suggest that sustainability should also be viewed as a change process involving adaptations and developments in response to the emerging needs of a system. Future research into sustainability of community mental health assets would benefit from exploring both definitions; planning research involving multiple timepoints would be a particularly important step to understanding more about sustainability as a process.

Given that most of the articles included in this review reported findings at the meso level (VSCE organisations), it would be useful to conduct further research into the micro and macro levels. Exploring some of the macro level factors that influence sustainability, such as higher level policies and funding is particularly important. Similarly, although this research provides insight into funding as a factor that affects sustainability, the cost‐effectiveness of community mental health assets has not been explored. With such a focus on funding, the cost‐effectiveness of these assets and their activities is a key part of the picture that requires further investigation.

## 7 CONCLUSIONS

The sustainability of community mental health assets is not yet well researched. Despite this, we identified a range of sustainability factors (both barriers and facilitators) at the micro, meso and macro levels which could help voluntary sector organisations and commissioners to improve mental health support in the community. Two key sustainability factors, connectedness and funding, were found at all three levels, highlighting the importance of these factors for maintaining the activities and benefits of VSCE organisations working to improve community mental health. Difficulty accessing and maintaining funding is not a new issue for the sustainability of community mental health assets, although this has been exacerbated by increased economic uncertainty and austerity policies in recent years. Yet if community mental health assets could be successfully sustained, they could offer important complementary support to the statutory sector and therefore reduce the burden on national health services. Whilst this review highlighted many factors affecting sustainability at the meso (organisational) level, further research into macro level factors, such as funding, is key to developing understanding of the sustainability of community mental health assets.

## AUTHOR CONTRIBUTIONS

A.M., M.B. and D.H. conceptualised the study, developed the design and methodology and conducted the literature screening. A.M. and A.H. completed the quality assessment of articles and A.M. conducted the data synthesis with support from M.B., D.H. and A.H. A.M. and M.B. prepared the original draft and D.H. and A.H. assisted with reviewing and editing the paper. All authors have read and agreed to the published version of the manuscript.

## FUNDING INFORMATION

This research was commissioned and funded by the MARCH Network, a national network funded by UK Research and Innovation (UKRI). The views expressed are those of the authors and not necessarily those of the MARCH Network.

## CONFLICT OF INTEREST

The authors state that there are no conflicts of interest.

## Supporting information


Appendix S1

## Data Availability

Data sharing is not applicable to this article as no new data were created or analysed in this study.

## References

[hsc13929-bib-0001] Arnstein, S. R. (1969). A ladder of citizen participation. Journal of the American Institute of Planners, 35(4), 216–224.

[hsc13929-bib-0002] Atif, N. , Bibi, A. , Nisar, A. , Zulfiqar, S. , Ahmed, I. , LeMasters, K. , Hagaman, A. , Sikander, S. , Maselko, J. , & Rahman, A. (2019). Delivering maternal mental health through peer volunteers: A 5‐year report. International Journal of Mental Health Systems, 13(1), 1–8. 31534475 10.1186/s13033-019-0318-3 PMC6747744

[hsc13929-bib-0003] Baxter, S. , Johnson, M. , Chambers, D. , Sutton, A. , Goyder, E. , & Booth, A. (2018). The effects of integrated care: A systematic review of UK and international evidence. BMC Health Services Research, 18(1), 1–13. 29747651 10.1186/s12913-018-3161-3 PMC5946491

[hsc13929-bib-0004] Cabinet Office . (2015). Building the big society . https://www.gov.uk/government/publications/building‐the‐big‐society

[hsc13929-bib-0057] Centre for Reviews and Dissemination . (2008). Systematic reviews: CRD's Guidance for Undertaking Reviews in Healthcare. University of York.

[hsc13929-bib-0005] Cheng, H. , Hayes, D. , Edbrooke‐Childs, J. , Martin, K. , Chapman, L. , & Wolpert, M. (2017). What approaches for promoting shared decision‐making are used in child mental health? A scoping review. Clinical Psychology & Psychotherapy, 24(6), O1495–O1511. 28752631 10.1002/cpp.2106

[hsc13929-bib-0006] Clapton, J. , Rutter, D. , & Sharif, N. (2009). SCIE systematic mapping guidance. SCIE.

[hsc13929-bib-0007] Coll‐Planas, L. , del Valle Gómez, G. , Bonilla, P. , Masat, T. , Puig, T. , & Monteserin, R. (2017). Promoting social capital to alleviate loneliness and improve health among older people in S pain. Health & Social Care in the Community, 25(1), 145–157. 26427604 10.1111/hsc.12284

[hsc13929-bib-0008] Department for Levelling Up, Housing and communities . (2021). Core spending power: Provisional local government finance settlement 2022 to 2023 . https://www.gov.uk/government/publications/core‐spending‐power‐provisional‐local‐government‐finance‐settlement‐2022‐to‐2023

[hsc13929-bib-0009] Di Gessa, G. , Maddock, J. , Green, M. J. , Thompson, E. J. , McElroy, E. , Davies, H. L. , Mundy, J. , Stevenson, A. J. , Kwong, A. S. F. , Griffith, G. J. , Katikireddi, S. V. , Niedzwiedz, C. L. , Ploubidis, G. B. , Fitzsimons, E. , Henderson, M. , Silverwood, R. J. , Chaturvedi, N. , Breen, G. , Steves, C. J. , … Patalay, P. (2021). Mental health inequalities in healthcare, economic, and housing disruption during COVID‐19: An investigation in 12 longitudinal studies. The British Journal of Psychiatry, 220(1), 21–30. 10.1192/bjp.2021.132 35045893

[hsc13929-bib-0010] Donnelly, K. Z. , Goldberg, S. , & Fournier, D. (2020). A qualitative study of LoveYourBrain yoga: A group‐based yoga with psychoeducation intervention to facilitate community integration for people with traumatic brain injury and their caregivers. Disability and Rehabilitation, 42(17), 2482–2491. 30741032 10.1080/09638288.2018.1563638

[hsc13929-bib-0011] Fan, Y. , Ma, N. , Ma, L. , Xu, W. , Lamberti, J. S. , & Caine, E. D. (2018). A community‐based peer support service for persons with severe mental illness in China. BMC Psychiatry, 18(1), 1–10. 29866096 10.1186/s12888-018-1763-2 PMC5987580

[hsc13929-bib-0012] Ferré, C. D. , Jones, L. , Norris, K. C. , & Rowley, D. L. (2010). The healthy African American families (HAAF) project: From community‐based participatory research to community‐partnered participatory research. Ethnicity & Disease, 20(102), 1–8. PMC3791221 20629240

[hsc13929-bib-0013] Fleisher, J. E. , Sennott, B. J. , Myrick, E. , Niemet, C. J. , Lee, M. , Whitelock, C. M. , Sanghvi, M. , Liu, Y. , Ouyang, B. , Hall, D. A. , Comella, C. L. , & Chodosh, J. (2020). KICK OUT PD: Feasibility and quality of life in the pilot karate intervention to change kinematic outcomes in Parkinson's disease. PLoS One, 15(9), e0237777. 32903267 10.1371/journal.pone.0237777 PMC7480843

[hsc13929-bib-0053] Foot, J. (2012). What makes us healthy. The asset approach in practice: Evidence, action, evaluation. http://janefoot.com/downloads/files/healthy%20FINAL%20FINAL.pdf

[hsc13929-bib-0014] Foster, A. , Thompson, J. , Holding, E. , Ariss, S. , Mukuria, C. , Jacques, R. , Akparido, R. , & Haywood, A. (2020). Impact of social prescribing to address loneliness: A mixed methods evaluation of a national social prescribing programme. Health & Social Care in the Community, 29(5), 1439–1449. 33084083 10.1111/hsc.13200

[hsc13929-bib-0054] Friedli, L. (2013). ‘What we've tried, hasn't worked’: The politics of assets based public health. Critical Public Health, 23(2), 131–145.

[hsc13929-bib-0015] Garven, F. , McLean, J. , & Pattoni, L. (2016). Asset‐based approaches: Their rise, role and reality. Dunedin Academic Press Ltd.

[hsc13929-bib-0016] Gorman, J. A. , McKay, C. E. , Yates, B. T. , & Fisher, W. H. (2018). Keeping clubhouses open: Toward a roadmap for sustainability. Administration and Policy in Mental Health and Mental Health Services Research, 45(1), 81–90. 27631611 10.1007/s10488-016-0766-x

[hsc13929-bib-0017] Gough, D. , Oliver, S. , & Thomas, J. (2017). An introduction to systematic reviews. Sage.

[hsc13929-bib-0018] Grant, G. , Machaczek, K. , Pollard, N. , & Allmark, P. (2017). Walking, sustainability and health: Findings from a study of a walking for health group. Health & Social Care in the Community, 25(3), 1218–1226. 28105761 10.1111/hsc.12424

[hsc13929-bib-0019] Hong, Q. N. , Pluye, P. , Fàbregues, S. , Bartlett, G. , Boardman, F. , Cargo, M. , Dagenais, P. , Gagnon, M.‐P. , Griffiths, F. , Nicolau, B. , O'Cathain, A. , Rousseau, M.‐C. , & Vedel, I. (2018). Mixed methods appraisal tool (MMAT). Canadian Intellectual Property Office, Industry Canada.

[hsc13929-bib-0058] Hopkins, T. , & Rippon, S. (2015). Head, hands and heart: Asset‐based approaches in health care. The Health Foundation.

[hsc13929-bib-0020] Institute for Government . (2021). Local government funding in England . https://www.instituteforgovernment.org.uk/explainers/local‐government‐funding‐england

[hsc13929-bib-0021] Kelly, D. , Steiner, A. , Mazzei, M. , & Baker, R. (2019). Filling a void? The role of social enterprise in addressing social isolation and loneliness in rural communities. Journal of Rural Studies, 70, 225–236. 31787802 10.1016/j.jrurstud.2019.01.024 PMC6876679

[hsc13929-bib-0022] Lennox, L. , Maher, L. , & Reed, J. (2018). Navigating the sustainability landscape: A systematic review of sustainability approaches in healthcare. Implementation Science, 13(1), 1–17. 29426341 10.1186/s13012-017-0707-4 PMC5810192

[hsc13929-bib-0023] Marmot, M. (2020). Health equity in England: The Marmot review 10 years on. BMJ, 368:m693. 32094110 10.1136/bmj.m693

[hsc13929-bib-0024] Martin, M. H. , Nielsen, M. B. D. , Petersen, S. M. , Jakobsen, L. M. , & Rugulies, R. (2012). Implementation of a coordinated and tailored return‐to‐work intervention for employees with mental health problems. Journal of Occupational Rehabilitation, 22(3), 427–436. 22246606 10.1007/s10926-011-9352-y

[hsc13929-bib-0025] Mental Health Foundation . (2017). Creating connections impact report 2014–2016. Mental Health Foundation.

[hsc13929-bib-0026] Mental Health Foundation . (2018). Young mums together report. Mental Health Foundation.

[hsc13929-bib-0027] Moore, A. , Stapley, E. , Hayes, D. , Town, R. , & Deighton, J. (2022). Barriers and facilitators to sustaining school‐based mental health and wellbeing interventions: A systematic review. International Journal of Environmental Research and Public Health, 19(6), 3587. 35329276 10.3390/ijerph19063587 PMC8949982

[hsc13929-bib-0028] Morgan, A. , & Ziglio, E. (2007). Revitalising the evidence base for public health: An assets model. Promotion and Education, 14(Suppl 2), 17–22. 17685075 10.1177/10253823070140020701x

[hsc13929-bib-0029] Munford, L. A. , Panagioti, M. , Bower, P. , & Skevington, M. S. (2020). Community asset participation and social medicine increases qualities of life. Social Science and Medicine, 259, 1131–1149. 10.1016/j.socscimed.2020.113149 PMC7397510 32603958

[hsc13929-bib-0030] Munford, L. A. , Sidaway, M. , Blakemore, A. , Sutton, M. , & Bower, P. (2017). Associations of participation in community assets with health‐related quality of life and healthcare usage: A cross‐sectional study of older people in the community. BMJ Open, 7(2), e012374. 10.1136/bmjopen-2016-012374 PMC5306503 28183807

[hsc13929-bib-0031] Naylor, C. , Parsonage, M. , McDaid, D. , Knapp, M. , Fossey, M. , & Galea, A. (2012). Long‐term conditions and mental health: The cost of co‐morbidities. The King's Fund.

[hsc13929-bib-0032] Ocloo, J. , & Maathews, R. (2016). From tokenism to empowerment: Progressing patient and public involvement in healthcare. BMJ Quality and Safety, 25, 626–632. 10.1136/bmjqs-2015-004839 PMC4975844 26993640

[hsc13929-bib-0033] Pace, R. , Pluye, P. , Bartlett, G. , Macaulay, A. C. , Salsberg, J. , Jagosh, J. , & Seller, R. (2012). Testing the reliability and efficiency of the pilot mixed methods appraisal tool (MMAT) for systematic mixed studies review. International Journal of Nursing Studies, 49(1), 47–53. 21835406 10.1016/j.ijnurstu.2011.07.002

[hsc13929-bib-0034] Page, M. J. , McKenzie, J. E. , Bossuyt, P. M. , Boutron, I. , Hoffmann, T. C. , Mulrow, C. D. , Shamseer, L. , Tetzlaff, J. M. , Akl, E. A. , Brennan, S. E. , Chou, R. , Glanville, J. , Grimshaw, J. M. , Hróbjartsson, A. , Lalu, M. M. , Li, T. , Loder, E. W. , Mayo‐Wilson, E. , McDonald, S. , … Moher, D. (2021). The PRISMA 2020 statement: An updated guideline for reporting systematic reviews. BMJ, 372, 1–11. 10.1136/bmj.n71 PMC8005924 33782057

[hsc13929-bib-0035] Palinkas, L. A. , Spear, S. E. , Mendon, S. J. , Villamar, J. , Reynolds, C. , Green, C. D. , Olson, C. , Adade, A. , & Brown, C. H. (2019). Conceptualizing and measuring sustainability of prevention programs, policies, and practices. Translational Behavioral Medicine, 10(1), 136–145. 10.1093/tbm/ibz170 PMC7020391 31764968

[hsc13929-bib-0036] Pieh, C. , Budimir, S. , Delgadillo, J. , Barkham, M. , Fontaine, J. R. , & Probst, T. (2021). Mental health during COVID‐19 lockdown in the United Kingdom. Psychosomatic Medicine, 83(4), 328–337. 33009276 10.1097/PSY.0000000000000871

[hsc13929-bib-0037] Pluye, P. , Potvin, L. , & Denis, J. L. (2004). Making public health programs last: Conceptualizing sustainability. Evaluation and Program Planning, 27(2), 121–133.

[hsc13929-bib-0038] Popay, J. , Roberts, H. , Sowden, A. , Petticrew, M. , Arai, L. , Rodgers, M. , Britten N. , Roen, K. , & Duffy, S. (2006). Guidance on the conduct of narrative synthesis in systematic reviews. *ESRC Methods Programme*.

[hsc13929-bib-0051] Rajkumar, R. P. (2020). COVID‐19 and mental health: A review of the existing literature. Asian Journal of Psychiatry, 52, 102066. 32302935 10.1016/j.ajp.2020.102066 PMC7151415

[hsc13929-bib-0039] Rutter, D. , Manley, C. , Weaver, T. , Crawford, M. J. , & Fulop, N. (2004). Patients or partners? Case studies of user involvement in the planning and delivery of adult mental health services in London. Social Science & Medicine, 58(10), 1973–1984. 15020013 10.1016/S0277-9536(03)00401-5

[hsc13929-bib-0056] Scheirer, M. A. (2005). Is sustainability possible? A review and commentary on empirical studies of program sustainability. American Journal of Evaluation, 26(3), 320–347.

[hsc13929-bib-0040] Schell, S. F. , Luke, D. A. , Schooley, M. W. , Elliott, M. B. , Herbers, S. H. , Mueller, N. B. , & Bunger, A. C. (2013). Public health program capacity for sustainability: A new framework. Implementation Science, 8(1), 1–9. 23375082 10.1186/1748-5908-8-15 PMC3599102

[hsc13929-bib-0041] Shields‐Zeeman, L. , Pathare, S. , Walters, B. H. , Kapadia‐Kundu, N. , & Joag, K. (2017). Promoting wellbeing and improving access to mental health care through community champions in rural India: The Atmiyata intervention approach. International Journal of Mental Health Systems, 11(1), 1–11. 28066505 10.1186/s13033-016-0113-3 PMC5210275

[hsc13929-bib-0042] Storm, M. , Hausken, K. , & Knudsen, K. (2011). Inpatient service providers' perspectives on service user involvement in Norwegian community mental health Centres. International Journal of Social Psychiatry, 57(6), 551–563. 20610463 10.1177/0020764010371270

[hsc13929-bib-0043] Suleman, M. , Sonthalia, S. , Webb, C. , Tinson, A. , Kane, M. , Bunbury, S. , Finch, D. , & Bibby, J. (2021). Unequal pandemic, fairer recovery: The COVID‐19 impact inquiry report. The Health Foundation.

[hsc13929-bib-0044] Thomas, J. , Graziosi, S. , Brunton, J. , Ghouze, Z. , O'Driscoll, P. , & Bond, M. (2020). EPPI‐reviewer: Advanced software for systematic reviews, maps and evidence synthesis. EPPI‐Centre software. UCL Social Research Institute.

[hsc13929-bib-0045] Van Bortel, T. , Wickramasinghe, N. D. , Morgan, A. , & Martin, S. (2019). Health assets in a global context: A systematic review of the literature. BMJ Open, 9(2), e023810. 10.1136/bmjopen-2018-023810 PMC6367963 30782888

[hsc13929-bib-0052] Vindegaard, N. , & Benros, M. E. (2020). COVID‐19 pandemic and mental health consequences: Systematic review of the current evidence. Brain, Behavior, and Immunity, 89, 531–542. 32485289 10.1016/j.bbi.2020.05.048 PMC7260522

[hsc13929-bib-0055] Wiltsey Stirman, S. , Kimberly, J. , Cook, N. , Calloway, A. , Castro, F. , & Charns, M. (2012). The sustainability of new programs and innovations: A review of the empirical literature and recommendations for future research. Implementation Science, 7(1), 1–19. 10.1186/1748-5908-7-17 PMC3317864 22417162

[hsc13929-bib-0046] Witte, S. S. , Burnette, D. , Aira, T. , & Myagmarjav, S. (2019). Global social welfare academic research partnerships: Lessons learned from two studies in Mongolia. Global Social Welfare, 6(3), 145–154.

