# Supplementary material for: Factors affecting the sustainability of community mental health assets: A systematic review
Source: Health Soc Care Community. 2022 Jul 28;30(6):e3369–83. doi: 10.1111/hsc.13929 (PMC10087471; doi:10.1111/hsc.13929)
Supplement: Supplementary file 1 — Appendix S1 [file HSC-30-e3369-s001.docx]

# Appendix A – Search Strategy

# Medline (Ovid)

## Sustainability

keyword:

(program* or intervention* or service* or provision* or therap*) adj5 (sustain* or continual or continuance or continues or maintain or maintenance or institutionali?ation or routini?ation or embed* or incorporation or integration or normali?ation or stabili?ation or durab* or ("long-term" or "long term" adj1 implement*) or discontinu* or mainstream* or "scale-up" or (scal* adj1 up) or "scaling-up" or endur* or persist*)

## Mental health issues

keyword:

(mental adj1 (disorder* or illness or health)) or (mental* adj1 fit*) or (emotional adj1 (problem* or adjustment or regulation or difficult* or illness* or disab* or disorder*)) or (affective adj1 disorder*) or (anxiety adj1 (disorder* or management or difficult*)) or anxiousness or (behavio?r adj1 (disorder* or problem* or difficult* or issue*)) or psychopathology or psychos* or psychotic or neuros* or neurotic or depress* or panic* or phobia* or (self adj1 harm*) or self-harm* or ((substance or drug or alcohol or eating) adj1 (difficult* or abuse or misuse or disorder* or issue*)) or bipolar* or anorexi* or bulimia* or overeat* or EDNOS or trauma* or PTSD or (("post traumatic" or post-traumatic) adj1 stress adj1 disorder*) or suicid* or delusion* or ((family or relationship* or peer*) adj1 (problem* or difficult* or disorder* or difficult* or issue*)) or internali* or externali*

## Service provision

keyword:

(intervention* or program* or sms or text or email* or app or apps or support or therap* or healing or class or classes or training) adj5 (wellbeing or "well being" or "well-being" or exercise or play or outdoor or sport* or walking or danc* or yoga or "tai chi" or pilates or "keep fit" or circuits or game* or gaming or social or non-clinical or "non clinical" or non-medical or "non medical" or art or relaxation or breathing or entertainment or reading or bibliotherapy or music or cinema or drama or religion or church or mosque or synagogue or pray* or faith* or mindfulness or spiritual* or meditat* or retreat or retreats or reflexology or reiki or massage or employment or volunteering or hobby or hobbies or diet or food or pets or creativ* or drawing or painting or garden* or horticulture or computer* or psychoeducation or "virtual reality" or holiday* or shopping or disabled or disabilit* or mobility)

or

fasciatherapy or hydrotherapy or aromatherapy or AA or NA or OA or (support adj group*) or ((light or wake) adj therap*) or (crystal adj healing) or (therapeutic adj communit*) or ((communit* or youth or social or lunch or carer*) adj2 (participation or group* or support or centre* or association* or club* or training)) or (collective adj action) or ((benefit* or housing or citizen* or legal) adj2 advice) or CAB or (("asset based" or asset-based or "strengths based" or strengths-based) adj approach*)

## Exclusions

NOT

keyword:

Anti-HIV Agents/ or Antiretroviral Therapy, Highly Active/ or antiretroviral therapy.mp. or Anti-Retroviral Agents/ or rats.mp or rats/ or (animal adj stud*) or (care adj home*)

or protocol (title only)

**Result:** 1230 hits, 1227 deduplicated.

# Web of Science

## Sustainability

topic:

(program* or intervention* or service* or provision* or therap*) near/5 (sustain* or continual or continuance or continues or maintain or maintenance or institutionali$ation or routini$ation or embed* or incorporation or integration or normali$ation or stabili$ation or durab* or long-term-implement* or discontinu* or mainstream* or scale-up or scaling-up or endur* or persist*)

## Mental health issues

topic:

(mental near/1 (disorder* or illness or health)) or (mental* near/1 fit*) or (emotional near/1 (problem* or adjustment or regulation or difficult* or illness* or disab* or disorder*)) or affective-disorder* or (anxiety near/1 (disorder* or management or difficult*)) or anxiousness or (behavio$r near/1 (disorder* or problem* or difficult* or issue*)) or psychopathology or psychos* or psychotic or neuros* or neurotic or depress* or panic* or phobia* or self-harm* or ((substance or drug or alcohol or eating) near/1 (difficult* or abuse or misuse or disorder* or issue*)) or bipolar* or anorexi* or bulimia* or overeat* or EDNOS or trauma* or PTSD or post-traumatic-stress-disorder* or suicid* or delusion* or ((family or relationship* or peer*) near/1 (problem* or difficult* or disorder* or difficult* or issue*)) or internali* or externali*

## Service provision

(intervention* or program* or sms or text or email* or app or apps or support or therap* or healing or class or classes or training) near/5 (wellbeing or well-being or exercise or play or outdoor or sport* or walking or danc* or yoga or tai-chi or pilates or keep-fit or circuits or game* or gaming or social or non-clinical or non-medical or art or relaxation or breathing or entertainment or reading or bibliotherapy or music or cinema or drama or religion or church or mosque or synagogue or pray* or faith* or mindfulness or spiritual* or meditat* or retreat or retreats or reflexology or reiki or massage or employment or volunteering or hobby or hobbies or diet or food or pets or creativ* or drawing or painting or garden* or horticulture or computer* or psychoeducation or virtual-reality or holiday* or shopping or disabled or disabilit* or mobility)

or

fasciatherapy or hydrotherapy or aromatherapy or AA or NA or OA or support-group* or ((light or wake) near/1 therap*) or crystal-healing or therapeutic-communit* or ((communit* or youth or social or lunch or carer*) near/2 (participation or group* or support or centre* or association* or club* or training)) or collective-action or ((benefit* or housing or citizen* or legal) near/2 advice) or CAB or ((asset-based or strengths-based) near/1 approach*)

## Exclusions

NOT

antiretroviral-therapy or rats or animal-stud* or care-home*

or protocol (title only)

## Limits

*AND***LANGUAGE:** (English) *AND* **DOCUMENT TYPES:** (Article  OR  Bibliography  OR  Book  OR  Book Chapter  OR  Discussion  OR  Early Access  OR  Proceedings Paper  OR  Review)

**Timespan:** 2010-2020. **Indexes:** SCI-EXPANDED, SSCI, CPCI-S, CPCI-SSH, BKCI-S, BKCI-SSH, ESCI.

2460 hits. Used Analyse function and excluded: Medicine general internal; clinical neurology; oncology; neuroscience; medicine research experimental; education special; criminology penology; infectious diseases; endocrinology metabolism; immunology; critical care medicine; rheumatology; surgery; obstetrics gynecology; gastroenterology hepatology; education scientific disciplines; engineering electrical electronic; urology nephrology; emergency medicine; engineering biomedical; anesthesiology; dentistry oral surgery medicine; biochemistry; computer science cybernetics; computer science engineering; otorhinolaryngology; veterinary science; biophysics; chemistry analytical.

Total: 1837, deduplicated 1833

# Websites

King’s fund: 19 hits. Scanned publications: topics: community services; mental health; new models of care; sustainability and transformation plans; voluntary and community sector; children and young people; health inequalities; older people; patient experience; access to care; commissioning and contracting; performance; productivity; social care finance.

Health Foundation: 2 hits. Scanned publications section.

Mind: 0 hits. No publications section. Searched website for community.

Age UK: 3 hits. Scanned reports and briefings, consultation responses, evaluation reports.

Sport England: 0 hits.

London Sport: 1 hit.

NCVO: 4. Scanned funding and finance; participation; public services; volunteering.

Work Foundation: 0 hits.

Nesta: 4. Scanned publications 2010-2020.

Joseph Rowntree foundation: 0 hits. Filtered by people/mental health.

Wellcome Collection: 0 hits. Searched 'mental health community' and mental health neighbourhood 2010-2020.

WHO: 0 hits.

Mental health foundation: 4.

Institute for Volunteering Research: 0 hits.

British Library social welfare portal: 10 hits. Scanned (2010-2020) Mental health services; Social care and social services; Community development and regeneration; Volunteering.

Social care online. 10 hits. Searched "mental health" and community and 2010-2020.

# Total

Deduplicated 2613
